# Supplementary material for: Bone need not remain an elephant in the room for radiocarbon dating
Source: R Soc Open Sci. 2021 Jan 13;8(1):201351. doi: 10.1098/rsos.201351 (PMC7890471; doi:10.1098/rsos.201351)
Supplement: Appendix A [file rsos201351supp1.html]

LATE-QUATERNARY MAMMAL BONE PRETREATMENT FOR RADIOCARBON DATING - EXPERT SURVEY - Google Forms   

JavaScript isn't enabled in your browser, so this file can't be opened. Enable and reload.

Untitled form

Send

Salvador Herrando-Perez

salherra@gmail.com

Manage your Google Account

Default

Salvador Herrando-Perez

salherra@gmail.com

All Brand accounts

Add another account

Sign out

Privacy Policy•Terms of Service

Untitled form

Questions

Responses

Section 1 of 4

Form title

LATE-QUATERNARY MAMMAL BONE PRETREATMENT FOR RADIOCARBON DATING - EXPERT SURVEY

Form description

THEME
+ This survey attempts to assess the importance of pretreatment of animal and human samples (bone, tooth, ivory) for radiocarbon dating from 50,000 radiocarbon years BP to Modern (Late Pleistocene to Present).
+ The survey can be completed in 5 to 10 minutes and has 4 sections as follows:
# section 1 (expertise) = 3 questions
# section 2 (pretreatment) = 4 questions
# section 3 (samples) = 3 questions
# section 4 (optional responses) = 2 questions
COMPLETION
+ To complete the survey, please click on the 'FILL OUT FORM' tab below.
+ You can revise your responses by clicking on 'Edit your response' \_after\_ submitting the survey. Please note that if you navigate back to a previous section before submitting the survey, your responses will be erased.
+ Kindly submit your responses by 15 December 2019.
ETHICS
The survey is strictly anonymous and has been approved by the Office of Research Ethics, Compliance and Integrity of the University of Adelaide (Australia) with Ethics Approval number H-2019-240. For any details, you can access the 'Participant Information Sheet' at https://drive.google.com/open?id=1xOWZo9IwxTerSyBUNDDv4pSnhjuc1bIc

Email address\*

Valid email address

Discipline that best describes YOUR EXPERTISE

\*

Question

Discipline that best describes YOUR EXPERTISE

\*

Question type

Short answer

Paragraph

Multiple choice

Checkboxes

Drop-down

File upload

Linear scale

Multiple-choice grid

Tick box grid

Date

Time

Description

Loading the image…

Caption

Anthropology

Archaeology

Biochemistry and Chemistry

Botany

Climatology

Ecology (Modern)

Ecology (Palaeoecology)

Evolutionary Biology

Genetics

Geochemistry

Geochronology

Geology

Marine Biology

Microbiology

Museum Studies (Curator, Collection Manager, Preparator)

Oceanography

Palaeontology

Physics

Zoology

Other…

Add option

or

Add "Other"

…

Answer key

(0 points)

Require a response in each rowRequiredRequired

Loading...

Loading…

TAXA your work/research mostly focuses on using radiocarbon measurements

\*

Question

TAXA your work/research mostly focuses on using radiocarbon measurements

\*

Question type

Short answer

Paragraph

Multiple choice

Checkboxes

Drop-down

File upload

Linear scale

Multiple-choice grid

Tick box grid

Date

Time

Description

Loading the image…

Caption

Animals

Humans

Both animals and humans

Other…

Add option

or

Add "Other"

…

Answer key

(0 points)

Require a response in each rowRequiredRequired

Loading...

Loading…

Have you ever worked at a radiocarbon DATING LABORATORY?

\*

Question

Have you ever worked at a radiocarbon DATING LABORATORY?

\*

Question type

Short answer

Paragraph

Multiple choice

Checkboxes

Drop-down

File upload

Linear scale

Multiple-choice grid

Tick box grid

Date

Time

Description

Loading the image…

Caption

YES

NO

Other…

Add option

or

Add "Other"

…

Answer key

(0 points)

Require a response in each rowRequiredRequired

Loading...

Loading…

After section 1

Continue to next section

Section 2 of 4

Section title (optional)

PRETREATMENT

Description (optional)

Please RANK each of the pretreatments below from 1 (weakly reliable) to 5 (strongly reliable) to REMOVE CONTAMINATION with exogenous carbon from a bone, tooth or ivory sample prior to radiocarbon dating?

\*

Question

Please RANK each of the pretreatments below from 1 (weakly reliable) to 5 (strongly reliable) to REMOVE CONTAMINATION with exogenous carbon from a bone, tooth or ivory sample prior to radiocarbon dating?

\*

Question type

Short answer

Paragraph

Multiple choice

Checkboxes

Drop-down

File upload

Linear scale

Multiple-choice grid

Tick box grid

Date

Time

Description

Please assume that the sample (1) includes 250 grams of starting material (bone, tooth or ivory) and (2) results in high collagen yield. Use the option 'I don't know / I am unsure' only if you didn't know or were unsure of how to rank any given pretreatment.

Loading the image…

Caption

I don't know / I am unsure

1 (weakly reliable)

2

3

4

5 (strongly reliable)

Hydroxyproline isolation from collagen gelatine

Ultrafiltration (>30 kDa) of collagen gelatine

XAD purification of collagen gelatine

Collagen gelatinization with none of the previous three pretreatments

Rows

1.

Hydroxyproline isolation from collagen gelatine

2.

Ultrafiltration (>30 kDa) of collagen gelatine

3.

XAD purification of collagen gelatine

4.

Collagen gelatinization with none of the previous three pretreatments

1.

Other…

5.

Add row

or

Add "Other"

Columns

I don't know / I am unsure

1 (weakly reliable)

2

3

4

5 (strongly reliable)

Other…

Add column

or

Add "Other"

…

Answer key

(0 points)

Require a response in each rowRequiredRequired

Loading...

Loading…

If you KNEW that a bone, tooth or ivory sample was SEVERELY CONTAMINATED with exogenous carbon, what pretreatment would you PREFER to be applied to the sample prior to radiocarbon dating?

\*

Question

If you KNEW that a bone, tooth or ivory sample was SEVERELY CONTAMINATED with exogenous carbon, what pretreatment would you PREFER to be applied to the sample prior to radiocarbon dating?

\*

Question type

Short answer

Paragraph

Multiple choice

Checkboxes

Drop-down

File upload

Linear scale

Multiple-choice grid

Tick box grid

Date

Time

Description

Please assume that (1) you have unlimited funding for radiocarbon dating, (2) the sample includes 250 grams of starting material (bone, tooth or ivory) and results in high collagen yield, and (3) the dating laboratory will return dating results to you within 1 week.

Loading the image…

Caption

Hydroxyproline isolation from collagen gelatine

Ultrafiltration (>30 kDa) of collagen gelatine

XAD purification of collagen gelatine

Gelatinization with none of the previous three pretreatments

I don't know / I am unsure

Other…

Add option

or

Add "Other"

…

Answer key

(0 points)

Require a response in each rowRequiredRequired

Loading...

Loading…

When SUBMITTING a bone, tooth or ivory sample to a radiocarbon dating laboratory, do you normally REQUEST a specific pretreatment method?

\*

Question

When SUBMITTING a bone, tooth or ivory sample to a radiocarbon dating laboratory, do you normally REQUEST a specific pretreatment method?

\*

Question type

Short answer

Paragraph

Multiple choice

Checkboxes

Drop-down

File upload

Linear scale

Multiple-choice grid

Tick box grid

Date

Time

Description

Loading the image…

Caption

YES

NO - pretreatment normally depends on the lab’s recommendations or experience

I have never submitted bone, tooth or ivory samples to a radiocarbon dating laboratory

Other…

Add option

or

Add "Other"

…

Answer key

(0 points)

Require a response in each rowRequiredRequired

Loading...

Loading…

When you COLLECT/USE a radiocarbon date of a bone, tooth or ivory sample FROM THE LITERATURE for your own research, have you ever considered pretreatment information as a criterion to rank its reliability?

\*

Question

When you COLLECT/USE a radiocarbon date of a bone, tooth or ivory sample FROM THE LITERATURE for your own research, have you ever considered pretreatment information as a criterion to rank its reliability?

\*

Question type

Short answer

Paragraph

Multiple choice

Checkboxes

Drop-down

File upload

Linear scale

Multiple-choice grid

Tick box grid

Date

Time

Description

Loading the image…

Caption

YES

NO

I have never collected/used radiocarbon dates from the literature

Other…

Add option

or

Add "Other"

…

Answer key

(0 points)

Require a response in each rowRequiredRequired

Loading...

Loading…

After section 2

Continue to next section

Section 3 of 4

Section title (optional)

SAMPLES

Description (optional)

Before SUBMITTING a bone, tooth or ivory sample to a radiocarbon dating laboratory, do you EXPECT or SUSPECT that the sample is CONTAMINATED with exogenous carbon?

\*

Question

Before SUBMITTING a bone, tooth or ivory sample to a radiocarbon dating laboratory, do you EXPECT or SUSPECT that the sample is CONTAMINATED with exogenous carbon?

\*

Question type

Short answer

Paragraph

Multiple choice

Checkboxes

Drop-down

File upload

Linear scale

Multiple-choice grid

Tick box grid

Date

Time

Description

Loading the image…

Caption

Always

Often

Sometimes

Never

I have never submitted a bone, tooth or ivory sample to a radiocarbon dating facility

Other…

Add option

or

Add "Other"

…

Answer key

(0 points)

Require a response in each rowRequiredRequired

Loading...

Loading…

Before EXTRACTING COLLAGEN from a bone, tooth or ivory sample prior to radiocarbon dating, do you EXPECT or SUSPECT that the sample is CONTAMINATED with exogenous carbon?

\*

Question

Before EXTRACTING COLLAGEN from a bone, tooth or ivory sample prior to radiocarbon dating, do you EXPECT or SUSPECT that the sample is CONTAMINATED with exogenous carbon?

\*

Question type

Short answer

Paragraph

Multiple choice

Checkboxes

Drop-down

File upload

Linear scale

Multiple-choice grid

Tick box grid

Date

Time

Description

Loading the image…

Caption

Always

Often

Sometimes

Never

I have never extracted collagen from a bone, tooth or ivory sample

Other…

Add option

or

Add "Other"

…

Answer key

(0 points)

Require a response in each rowRequiredRequired

Loading...

Loading…

Please RANK from lowest (rank = 1) to highest (rank = 5) importance each of the following CRITERIA to choose pretreatment before submitting bone, tooth or ivory samples to a radiocarbon dating laboratory.

\*

Question

Please RANK from lowest (rank = 1) to highest (rank = 5) importance each of the following CRITERIA to choose pretreatment before submitting bone, tooth or ivory samples to a radiocarbon dating laboratory.

\*

Question type

Short answer

Paragraph

Multiple choice

Checkboxes

Drop-down

File upload

Linear scale

Multiple-choice grid

Tick box grid

Date

Time

Description

If you have never submitted bone, tooth or ivory samples to a radiocarbon dating facility, please give rank = 1 to all criteria.

Loading the image…

Caption

1 (lowest importance)

2

3

4

5 (highest importance)

Available amount of bone per sample

Price per sample

Research question under investigation

I let the dating lab decide

I choose pretreatment offered only by dating facilities with high international reputation

Geographical origin of the bones

Study species

A priori knowledge of contamination

Journal/Book/Report I am going to publish the C14 dates in

Return time of dating results from the dating facility

Rows

1.

Available amount of bone per sample

2.

Price per sample

3.

Research question under investigation

4.

I let the dating lab decide

5.

I choose pretreatment offered only by dating facilities with high international reputation

6.

Geographical origin of the bones

7.

Study species

8.

A priori knowledge of contamination

9.

Journal/Book/Report I am going to publish the C14 dates in

10.

Return time of dating results from the dating facility

1.

Other…

11.

Add row

or

Add "Other"

Columns

1 (lowest importance)

2

3

4

5 (highest importance)

Other…

Add column

or

Add "Other"

…

Answer key

(0 points)

Require a response in each rowRequiredRequired

Loading...

Loading…

After section 3

Continue to next section

Section 4 of 4

Section title (optional)

FEEDBACK

Description (optional)

(Optional response) Please CITE one RESEARCH PAPER that you would cite to support your choice of the most reliable pretreatment of a bone, tooth or ivory sample for radiocarbon dating

\*

Question

(Optional response) Please CITE one RESEARCH PAPER that you would cite to support your choice of the most reliable pretreatment of a bone, tooth or ivory sample for radiocarbon dating

\*

Question type

Short answer

Paragraph

Multiple choice

Checkboxes

Drop-down

File upload

Linear scale

Multiple-choice grid

Tick box grid

Date

Time

Description

Please use either doi (example = https://doi.org/10.1126/science.114.2960.291) or ' firstAuthor Year Journal Volume-firstPage ' (example = Johnson 1959 Radiocarbon 1-199)

Loading the image…

Caption

Long-answer text

Length

Regular expression

Maximum character count

Minimum character count

Number

Custom error text

Answer key

(0 points)

Require a response in each rowRequiredRequired

Loading...

Loading…

(Optional response) Please feel free to MAKE COMMENTS about (i) the content or format of this survey and/or (ii) your experience in dating bone, tooth or ivory samples

\*

Question

(Optional response) Please feel free to MAKE COMMENTS about (i) the content or format of this survey and/or (ii) your experience in dating bone, tooth or ivory samples

\*

Question type

Short answer

Paragraph

Multiple choice

Checkboxes

Drop-down

File upload

Linear scale

Multiple-choice grid

Tick box grid

Date

Time

Description

Loading the image…

Caption

Long-answer text

Length

Regular expression

Maximum character count

Minimum character count

Number

Custom error text

Answer key

(0 points)

Require a response in each rowRequiredRequired

Loading...

Loading…

The number of responses collected by this form may exceed the limit supported by Sheets. If you're having trouble viewing your responses in Sheets, try downloading a .CSV instead.

Not accepting responses

Accepting responses

Message for respondents

This form is no longer accepting responses

Summary

Question

Individual

Insights

Total points distribution

Loading...

Loading responses…

Discipline that best describes YOUR EXPERTISE

No responses yet for this question.

TAXA your work/research mostly focuses on using radiocarbon measurements

No responses yet for this question.

Have you ever worked at a radiocarbon DATING LABORATORY?

No responses yet for this question.

PRETREATMENT

Please RANK each of the pretreatments below from 1 (weakly reliable) to 5 (strongly reliable) to REMOVE CONTAMINATION with exogenous carbon from a bone, tooth or ivory sample prior to radiocarbon dating?

No responses yet for this question.

If you KNEW that a bone, tooth or ivory sample was SEVERELY CONTAMINATED with exogenous carbon, what pretreatment would you PREFER to be applied to the sample prior to radiocarbon dating?

No responses yet for this question.

When SUBMITTING a bone, tooth or ivory sample to a radiocarbon dating laboratory, do you normally REQUEST a specific pretreatment method?

No responses yet for this question.

When you COLLECT/USE a radiocarbon date of a bone, tooth or ivory sample FROM THE LITERATURE for your own research, have you ever considered pretreatment information as a criterion to rank its reliability?

No responses yet for this question.

SAMPLES

Before SUBMITTING a bone, tooth or ivory sample to a radiocarbon dating laboratory, do you EXPECT or SUSPECT that the sample is CONTAMINATED with exogenous carbon?

No responses yet for this question.

Before EXTRACTING COLLAGEN from a bone, tooth or ivory sample prior to radiocarbon dating, do you EXPECT or SUSPECT that the sample is CONTAMINATED with exogenous carbon?

No responses yet for this question.

Please RANK from lowest (rank = 1) to highest (rank = 5) importance each of the following CRITERIA to choose pretreatment before submitting bone, tooth or ivory samples to a radiocarbon dating laboratory.

No responses yet for this question.

FEEDBACK

(Optional response) Please CITE one RESEARCH PAPER that you would cite to support your choice of the most reliable pretreatment of a bone, tooth or ivory sample for radiocarbon dating

No responses yet for this question.

(Optional response) Please feel free to MAKE COMMENTS about (i) the content or format of this survey and/or (ii) your experience in dating bone, tooth or ivory samples

No responses yet for this question.

Waiting for responses

of

15

of

1

Google Account

Salvador Herrando-Perez

salherra@gmail.com

.
