# Supplementary material for: Bone need not remain an elephant in the room for radiocarbon dating
Source: R Soc Open Sci. 2021 Jan 13;8(1):201351. doi: 10.1098/rsos.201351 (PMC7890471; doi:10.1098/rsos.201351)
Supplement: Appendix C [file rsos201351supp3.docx]

**Bone need not remain an elephant in the room for radiocarbon dating**

**Salvador Herrando-Pérez**

**Supplemental material / Appendix C**

(figures S1 to S4 and tables S1 to S2)

**Figure S1. Respondent profiles.** Three descriptors qualifying the profile of the target audience (132 respondents from 25 countries) by their (a) study taxa (survey question 1.2), (b) experience working in a radiocarbon (^14^C) laboratory (survey question 1.3), and (c) research expertise (survey question 1.1). Respondent proportions by country and expertise presented below.

**Figure S2. Do respondents have experience handling bone samples for radiocarbon dating?** Proportion of target audience (132 respondents) who has (a) submitted samples of raw bone to a radiocarbon ^14^C laboratory (*n* = 116*, survey question 3.1) or (b) extracted the collagen gelatine (*n* =105**, survey question 3.2) for ^14^C dating.

**Figure S3. Is pretreatment considered as a quality criterion of radiocarbon dates of bone collated from the literature?** Proportion of 113 respondents (of a target audience of 132) who collates bone radiocarbon dates from the literature for their own research and does (‘YES’) or does not (‘NO’) consider bone pretreatment as an indicator of data quality (survey question 2.4). ‘No user’ indicates respondents who have never collated bone radiocarbon dates from the literature for their own research (*n =* 4, 4% of the target audience).

**Figure S4. Who chooses bone pretreatment prior to radiocarbon dating?** Proportion of respondents (*n =* 113 of 132*) that choose pretreatment themselves or ask the radiocarbon (^14^C) laboratory to choose pretreatment for them when submitting a bone sample to a ^14^C laboratory (survey question 2.3).

**Table S1.** **Publications* that 46 respondents (of a target audience of 132) would cite to support their choice of the most reliable bone pretreatment for ^14^C dating** (survey question 4.1).

**Table S2. Literal feedback provided by 50 respondents (of a target audience of 132) about the content/format of the survey and/or their experience in radiocarbon (^14^C) dating bone samples** (survey question 4.2). ‘# Respondent’ represents a unique numerical identifier assigned to each respondent. The text has been curated for abbreviation, conceptual references, literature citations, punctuation and typos, while both curated and non-curated text is available in Appendix B. The identity of the respondents is unknown (see subsection ‘Ethical Statement’ in Materials and Methods).

**Figure S1. Respondent profiles.** Three descriptors qualifying the profile of the target audience (132 respondents from 25 countries) by their (a) study taxa (survey question 1.2), (b) experience working in a radiocarbon (^14^C) laboratory (survey question 1.3), and (c) research expertise (survey question 1.1). Respondent proportions by country and expertise presented below*.

| 1. *Study taxa* | 1. *Experience in ^14^C lab* | 1. *Research expertise* |
| --- | --- | --- |
|  |  |  |

*

*Countries:* Argentina = 2 respondents (<2%), Australia = 12 (9%), Belgium = 2 (<2%), Brazil = 1 (<1%), Canada = 10 (8%), Chile = 1 (<1%), China = 4 (3%), Denmark = 3, Finland = 1 (<1%), France = 2 (<2%), Germany = 12 (9%), Hungary =1 (<1%), Italy = 4 (3%), Japan = 1 (<1%), Netherlands =3, New Zealand = 6 (5%), Poland = 4 (3%), Portugal = 1 (<1%), Romania = 1 (<1%), Russia = 3 (2%), Spain = 2 (<2%), Sweden = 3, Switzerland 1, UK: England = 13 / Northern Ireland = 1 / Scotland = 2 (12%), USA = 36 (27%).

*Expertise:* Proportion of respondents among all disciplines (in increasing order of frequency) were archaeology (*n* = 44, 33% of 132), geochronology (*n* = 22, 17%), palaeontology (*n* = 15, 11%), biochemistry (*n* = 12, 9%), geochemistry (*n* = 7, 5%), physics (*n* = 9, 7%), evolutionary biology (*n* = 5, 4%), palaeoecology (*n* = 5, 4%), genetics (*n* = 4, 3%), anthropology (*n* = 4, 3%), geology (*n* = 3, 2%), climatology (*n* = 1, 1%) and oceanography (*n* = 1, 1%).

**Figure S2. Do respondents have experience handling bone samples for radiocarbon dating?** Proportion of target audience (132 respondents) who has (a) submitted samples of raw bone to a radiocarbon ^14^C laboratory (*n* = 116*, survey question 3.1) or (b) extracted the collagen gelatine (*n* =105**, survey question 3.2) for ^14^C dating.

| \| 1. *Submitting raw bone* \| \| --- \| \| ** \|  1. *Extracting collagen gelatine* |
| --- | --- | --- |
| ****** |

*Excluding 16 respondents who stated not to have submitted samples of raw bone to a ^14^C lab (figure 3).

**Excluding 27 respondents who stated not to have extracted collagen gelatine.

**Figure S3. Is pretreatment considered as a quality criterion of radiocarbon dates of bone collated from the literature?** Proportion of 113 respondents (of a target audience of 132) who collates bone radiocarbon dates from the literature for their own research and does (‘YES’) or does not (‘NO’) consider bone pretreatment as an indicator of data quality (survey question 2.4). ‘No user’ indicates respondents who have never collated bone radiocarbon dates from the literature for their own research (*n =* 4, 4% of the target audience).

| ****** |
| --- |

**Figure S4. Who chooses bone pretreatment prior to radiocarbon dating?** Proportion of respondents (*n =* 113 of 132*) that choose pretreatment themselves or ask the radiocarbon (^14^C) laboratory to choose pretreatment for them when submitting a bone sample to a ^14^C laboratory (survey question 2.3).

| ****** |
| --- |

*Excluding 17 respondents who never submitted bone to a ^14^C laboratory.

**Table S1.** **Publications* that 46 respondents (of a target audience of 132) would cite to support their choice of the most reliable bone pretreatment for ^14^C dating** (survey question 4.1).

| **Reference (citation # number)** | **Hits** | **# Respondent** | **Pretreatment** | **ORAU** |
| --- | --- | --- | --- | --- |
| Beaumont *et al.* [1] | 1 | 111 | hyp | no |
| Becerra-Valdivia *et al.* [2] | 2 | 5, 26 | hyp | yes |
| Bird *et al.* [3] | 1 | 126 | uf | no |
| Boudin *et al.* [4] | 1 | 7 | none | no |
| Brock *et al.* [5] | 2 | 64, 91 | hyp | yes |
| Brock *et al.* [6] | 3 | 65, 83, 104 | hyp, uf, none | yes |
| Brock *et al.* [7] | 1 | 128 | hyp | yes |
| Brock *et al.* [8] | 3 | *7*, *46*, 127 | none, hyp, gel | yes |
| Brown *et al.* [9] | 2 | *11, 32* | uf | no |
| Bruhn *et al.* [10] | 1 | 101 | hyp | no |
| Cersoy *et al.* [11] | 1 | 33 | hyp | no |
| Cook *et al.* [12] | 1 | 53 | uf | yes |
| DeNiro [13] | 1 | 97 | uf | no |
| Devièse *et al.* [14] | 2 | 48, 99 | hyp | yes |
| Devièse *et al.* [15] | 5 | 4, 19, 51, 69, 70 | hyp | yes |
| Dinnis *et al.* [16] | 1 | 119 | hyp | yes |
| Dunbar *et al.* [17] | 1 | 85 | none | no |
| Dunbar *et al.* [18] | 1 | 122 | hyp | no |
| Gillespie *et al.* [19] | 1 | 13 | hyp | yes |
| Gilmour *et al.* [20] | 1 | 22 | hyp | yes |
| Higham *et al.* [21] | 2 | 68, 70 | hyp, uf | yes |
| Higham [22] | 1 | 99 | hyp | yes |
| Hüls *et al.* [23] | 1 | 101 | hyp | no |
| Hüls *et al.* [24] | 1 | 101 | hyp | no |
| Huels *et al.* [25] | 1 | 16 | hyp | yes |
| Jacobi *et al.* [26] | 1 | 108 | hyp | yes |
| Kosintsev *et al.* [27] | 1 | 36 | hyp | yes |
| Kuzmin [28] | 1 | *11* | uf | no |
| Longin [29] | 1 | *99* | hyp | yes |
| Marom *et al.* [30] | 2 | 74, 112 | hyp | yes |
| McDonald *et al.* [31] | 1 | 21 | xad | no |
| Politis *et al.* [32] | 1 | 105 | hyp | no |
| Potter *et al.* [33] | 1 | 3 | uf | no |
| Redmond *et al.* [34] | 1 | 124 | xad | no |
| Stafford *et al.* [35] | 1 | 23 | xad | no |
| Tisnérat-Laborde *et al.* [36] | 1 | 69 | hyp | no |
| van Klinken [37] | 2 | 67, 98 | hyp | yes |
| Waterbolk [38] | 1 | 10 | uf | no |
| Waters and Stafford [39] | 1 | 42 | xad | no |
| Waters *et al.* [40] | 1 | 77 | xad | no |
| Zazula *et al.* [41] | 2 | 93, 103 | hyp | yes |

*‘Hits’ shows the number of times a publication was suggested, ‘# Respondent’ is an unique numerical identifier assigned to each respondent (*numbers in italics indicate respondents who cited a reference to describe their experience in dating bone* [survey question 4.2; table S2]), ‘Pretreatment’ indicates the bone pretreatment that each respondent selected as the most reliable, and ‘ORAU’ represents whether the papers suggested included (‘yes’) or excluded (‘no’) at least one co-author affiliated with the research centre hosting the University of Oxford’s Radiocarbon Accelerator Unit. Abbreviations: gel = gelatinization without further pretreatment, hyp = hydroxyproline isolation, uf = ultrafiltration, xad = XAD-2 purification, none = respondents who chose no pretreatment (figure 2).

**Table S2. Literal feedback provided by 50 respondents (of a target audience of 132) about the content/format of the survey and/or their experience in radiocarbon (^14^C) dating bone samples** (survey question 4.2). ‘# Respondent’ represents a unique numerical identifier assigned to each respondent. The text has been curated for abbreviation, conceptual references, literature citations, punctuation and typos, while both curated and non-curated text is available in Appendix B. The identity of the respondents is unknown (see subsection ‘Ethical Statement’ in Materials and Methods). Each response has been classified into 13 non-exclusive categories according to ‘content types’ as follows:

- ***Accuracy*** = views about the improvement of the accuracy of ^14^C data.
- ***Alkali*** = use of alkaline rinses as part of bone pretreatment.
- ***A priori assessment*** = assessment of contamination sources and types prior to ^14^C dating of skeletal material.
- ***^14^C laboratory*** = aspects concerning the communication or commercial exchange of users of ^14^C data with ^14^C laboratories.
- ***Conservation substance*** = pretreatment steps to remove conservation substances from museum material.
- ***Caveat*** = caveats respondents experienced to answer some questions in the survey.
- ***Depositional context* =** interpretation of ^14^C data relative to the taphonomy of the skeletal material being dated.
- ***Survey importance =*** comments highlighting the importance of the survey.
- ***Pretreatment merit*** *=* views about the relative merits of one or several pretreatments
- ***Quality indicator*** *=* use of chemical indicators of reliability of ^14^C dates (e.g., C[carbon]:N[nitrogen] ratios).
- ***Reference material*** = use of standard reference materials to evaluate the reliability of ^14^C data.
- ***Reporting etiquette*** = clarity and completeness of ^14^C data reported in publications.
- ***Sample specificity*** = aspects of the age, amount, chemistry or structural integrity of samples of skeletal materials for ^14^C dating.

| **#**  **Respondent** | **Feedback** | **Content type** |
| --- | --- | --- |
| **1** | I have never had much to do with these sorts of samples, so can’t really comment very authoritatively. | Caveat |
| **2** | I tend to only trust laboratories where you can have dialogue with technicians to discuss the context of samples and best options for pretreatment. This is not always possible with low-cost high-throughput commercial labs, and the quality of the dates they produce is therefore likely to suffer. | ^14^C laboratory |
| **3** | The most important criterion, far more important than pretreatment, and one that is often not considered (as exemplified in this survey) is ‘context’ of the specimen. That is, clear and unambiguous control of association and context of the sample with respect to the cultural activities in question. This criterion is best evaluated by the excavator (P.I.) in the field at the time of recovery, with full knowledge of the stratigraphic, 3-dimensional context of the sample. | Depositional context |
| **4** | If AMS labs spent as much money on chemistry and biology as they do on physics, the inherent inaccuracy in most bone ^14^C dates would have been eliminated years ago. | Accuracy |
| **7** | The pretreatment can depend on the type of contamination. If the sample is contaminated with PVA [PVA jargon revised by Brock,Dee,Hughes,Snoeck,Staff and Bronk Ramsey [8]] (for conservation), solvents are the solution before collagen extraction. But if the sample is contaminated with humic acids, NaOH wash can remove them (the NaOH-step was not mentioned in this survey). You can not choose the best method. In s[o]me cases, ultrafiltration is better in others not. | Alkali  *A priori* assessment  Conservation substance  Pretreatment merit |
| **10** | Accuracy is crucial, that goes without saying. However, in the rush to push ^14^C dating beyond its reasonable limit of applicability, there has been a tendency to overlook that accuracy is just the beginning. Ultimately, any interpretation of a ^14^C date depends on issues of association. Waterbolk [38] explained it very clearly almost 50 years ago, and that is why I choose to cite his paper as the provider of the framework that arch[a]eologists and dating experts alike must bear in mind when making decisions, including ones on choice of pretreatment (e.g., why date a bone to begin with, if, no matter how advanced the pretreatment technique and how very certain we can be of the accuracy of the measurement, we can’t be really sure what the ‘date’ means for the issue that the dating is supposed to tackle?) | Accuracy  Depositional context |
| **11** | The standard and easiest pretreatment of bone would be the >30 kDa method developed by Brown,Nelson,Vogel and Southon [9]. But one needs to consider the context and age of the bones. For poorly degraded bones, you need to use XAD purification or hydroxyproline dating. But hydroxyproline dating is far too expensive for my individuals unless they have a very rare specimen. Thus, XAD purification is cheaper and does a good job. For bones that have been conserved, one needs to use a series of solvent extractions to get rid of the contaminants. Bones are fairly easy to assess for dates as one can use the C:N ratio and %C and %N to assess the amount of contamination, and thus reliability of the dates — if you have experience in this area. This survey is deeply naive. In my opinion NONE of the methods listed (Longin [gelatinization alone], ultrafiltration, hydroxyproline isolation, or XAD purification) are entirely reliable. XAD purification and hydroxyproline isolation both suffer from potential column bleed, ultrafiltration has well documented risks of adding contaminants (and in my experience can also concentrate natural contaminants), Longin is probably good enough most of the time for well preserved Holocene bone (and my experience is that some laboratories are much better at getting accurate results using Longin variants than others, for reasons that are not clear to me). I am not particularly interested in dating pre-Holocene bone; I am very interested in high-precision (2 per mill; yes, that is +/-16 or better!) AMS dating of Holocene bone. In my opinion no one is anywhere near that yet (John Southon [https://www.ess.uci.edu/people/jsouthon], who ultrafilters, is probably closest, but until someone else can replicate him at the same precision, the jury is out!). Ideally replicate using a different method. Otherwise you are sunk in the ‘it is older, it is better’ argument (which, I agree with Kuzmin [28], stinks!). The bottomline problem here is the lack of a range of known-age standards that are available in sufficient quantity for all laboratories to use. Such known-age material that there is (Mary Rose bone, the 7-years war horse) is simply not available in sufficient quantity, and anyway are young and well-preserved enough that all the methods listed get accurate ages. No one is questioning that all of these methods most of the time remove 99% of the contaminants. Whether that is good enough at 30,000 years BP, and whether that is good enough at +/-16, is another matter. Another issue that this survey did not cover, but which is in my view critical, is the use of a whole-process bone blank in calculating ^14^C ages for bone samples whichever method of pretreatment is chosen. In my experience, less than 1/3 of ^14^C laboratories use such a standard — this is frightening. I answered ‘Ultrafiltration’ for question 2.2 (Pretreatment) — this was only so I could continue with the survey. The real answer is ‘none of the above’. | Accuracy  Caveat  Depositional context  Pretreatment merit  Quality indicator  Reference material  Sample specificity |
| **12** | This survey is far too general, and bones need to be dated using the correct pretreatment on a case-by-case basis. There is not a one pretreatment that fits all types of specimens. Still, the >30 kDa UF method is the easiest, cheapest and most reliable for let’s say 80% of the specimens that come through a typical ^14^C laboratory. | Caveat  Pretreatment merit  Sample specificity |
| **13** | This is a little tricky for someone like me who has worked in ^14^C laboratories since 1970, where I mainly did the chemistry and dating, not the submitting. These materials have always been difficult in the lab, because they are always contaminated and often have only small amounts of protein for dating. Later, when I chose laboratories based on availability and/or funding, ORAU’S ultrafiltration method [https://c14.arch.ox.ac.uk] was the best on offer, but in my experience this did not always work satisfactorily. If the amount of material is sufficient, and money is not a problem, isolating hydroxyproline for dating is the best option (and I was the first to actually publish single amino acid dates), but there are further possibilities I would like to explore given adequate funding. | Caveat  ^14^C laboratory  Pretreatment merit  Sample specificity |
| **16** | In my opinion, the biggest drawback to ^14^C research is the lack of pretreatment information and quality criteria published alongside ^14^C dates, so that the reliability of the dates cannot be established by other researchers. This renders much of the published data very unhelpful. | Quality indicator  Reporting etiquette |
| **23** | I am surprised to see that there are not any questions about using C:N ratios. | Caveat  Quality indicator |
| **26** | Despite my answers to your survey, I remain unconvinced that any pretreatment method is better than any other. If I’m not mistaken, that was a conclusion of the last International Radiocarbon Intercomparison. | Pretreatment merit |
| **27** | I have had a good amount of experience with various ^14^C labs. There are only a few I trust based on results but, with the labs I trust, I always follow their recommendations. | ^14^C laboratory |
| **28** | I don’t believe there is one ‘best method’. The different methods each balance strengths and weaknesses, advantages and disadvantages. Different burial environments result in different collagen chemistries, and introduce different contaminants. A method that works well in one circumstance might not be optimal in another. My experience is also that elaborate methods tend to have advantages when preservation is poor. If bones are well preserved, and collagen yields are high, often there is no statistically meaningful difference between simple and elaborate treatments. I think you should also carefully consider how often it is actually possible to identify what the ‘correct’ answer is because, unlike with wood, reliably known-age collagen-containing materials are very difficult to obtain. Without known-age controls, meaningful comparison of each method’s ability to give the right answer are not possible. So one is left with assessment methods based upon repetition and intercomparison, or assumptions such as the picking method that generates the oldest date. But these approaches don’t guarantee a correct answer. | Accuracy  Depositional context  Pretreatment merit  Reference material  Sample specificity |
| **32** | Before sending any samples for ^14^C dating, I would extract the collagen myself following the modified Longin method from Brown,Nelson,Vogel and Southon [9]. If I am to deal with modern marine /freshwater specimens I would extract lipids (chloroform:methanol) from the samples — even though it may not affect the dating of them. | Sample specificity  Pretreatment merit |
| **37** | Very important survey! Thank you very much. | Survey importance |
| **38** | I haven’t been able to answer many of the questions, as I worked in a ^14^C lab but have never submitted bones (other than ones I was going to pretreat myself in my own lab!). However, when I lecture about the practicals of ^14^C dating (especially to students/practitioners in forensic archaeology/anthropology) I cover how to select a lab, and make it clear that I might pick different labs for different samples. For example, I would always recommend a ‘research lab’ (i.e., affiliated to a university, and with a good record of method development for that material) for older and/or potentially contaminated bones, but if you’re an archaeologist just needing to confirm the age of a load of bones recently excavated prior to road building on top of site, for example, then one of the quicker/cheaper commercial labs will be sufficient. I also advise that there is no evidence to suggest that ultrafiltration makes a difference to younger (<25,000 years BP) bones, and so it’s not necessary to select/pay extra for this option if the bones are known to be much more recent. I also suggest people consider how long they want their samples to be kept for afterwards — research labs will often store leftover material (treated and untreated) indefinitely, but commercial labs don’t always, and so may not be able to help with queries after a relatively short time period after dating. Mark van Strydonck [http://www.kikirpa.be] used to have an excellent cartoon that he included in conference presentations showing how you got what you paid for in terms of ^14^C dating, including a gypsy staring into her crystal ball — I always wanted a copy of that for my teaching, as I think it’s scarily true, and too many people think that ‘a ^14^C date is a ^14^C date’. Unfortunately, ivory is a difficult material to cover in a questionnaire like this — for older ivory excavated by archaeologists/palaeontologists (especially if well-preserved from the permafrost) then a commercial ^14^C lab may be fine. But there is a separate community interested in ^14^C dates on ivory, i.e. museums, art dealers, auction houses, etc., who really need well-established ^14^C laboratories who can often assist with sampling valuable objects when dating to establish whether the ivory can be traded legally or not, or to comment on the object’s authenticity. These objects can often only provide a very small amount of material, so you need a lab who can confidently sample enough material to provide a date, while using as little material as possible, to avoid needless destruction of an artefact. | Accuracy  Caveat  ^14^C laboratory  Pretreatment merit  Sample specificity |
| **40** | The options given in this survey are far too simple in my opinion. The ^14^C community in general often assumes there is a perfect pretreatment method for a given sample type. In my experience, it is often more promising to determine the kind of contamination more precisely and apply a specific cleaning method, e.g. finding a proper solvent for a varnished sample or ensuring proper removal of larger amounts of humic acids by additional alkali steps. For the effort a ^14^C measurement is requiring, every sample deserves the best individual pretreatment. Always consider possibly unneeded pretreatment steps might introduce foreign carbon to your sample. | Caveat  Conservation substance  Pretreatment merit  Sample specificity |
| **42** | In comparing results by splitting samples for XAD purification and ultrafiltration, XAD purification performed better in matching age provided by charcoal and projectile point type for Folsom sites on Southern Plains of North America. | Pretreatment merit |
| **44** | I find that projects that date different fractions of bone samples to gauge source of contamination and the success of its subsequent removal offer the most reliable age estimates. For instance, if a study dates the Acid-Base-Acid collagen and ultrafiltered collagen, and may even choose to do some direct dating of proteins, then the age of that specimen is much more reliable since the source and degree of contamination is clear. | Alkali  *A priori* assessment  Pretreatment merit  Sample specificity |
| **45** | Since I run a ^14^C lab (and I suspect this is also true for a significant fraction of the people contacted for this survey), many of the questions about sample submission as ph[r]ased are impossible to answer sensibly. Also, some of the conditions that are set up for the questions are completely impractical, e.g., for the questions on section 2 (Pretreatment) show me one single ^14^C lab on the entire planet that could return an XAD purification or hydroxyproline date in one week. That’s significant: does ‘most reliable’ in the optional questions above mean removing all likely contamination in 99.9% of all cases vs in 99.5% or 99.0% or 95.0%; or does it mean something that works in well over 95.0% of all cases with reasonable turnaround *versus* having to wait six months? Third, the question about ‘do you suspect or expect’ contamination misses the point, which is that, regardless of what you suspect, you ALWAYS treat the sample as if it’s contaminated. Even the very simplest gelatinization procedure is specifically designed to remove soil carbon contamination, and if done correctly, Maillard reaction contaminants as well. The lengths to which one goes to remove that contamination depend on estimates (=guesses) of what the contamination is and how bad, and also on the context of the sample — how important is it? (is it the human skeleton found next to the *Diprotodon* that has already been dated to >50,000 years BP?), has it already yielded a weird date?, etc. | Accuracy  *A priori* contamination  Caveat  Depositional context  ^14^C laboratory  Pretreatment merit |
| **46** | I found some of this very difficult to answer given that I am the ^14^C lab (I hope I’ve been consistent and what follows is not too pedantic). The effect of age of the sample is extremely important in terms of deciding pretreatment and expected effect of the contamination, e.g., I would consider doing a simple Acid-Base-Acid-gelatinization on rodent bones too small for other methods if they were only a few hundred years old (where we know ultrafiltration does nothing but add the possibility for lab contamination), but would not do this if they were Pleistocene as the effect of any contaminant is so much higher for older samples. In terms of the role of research question — this is also crucial, but depends heavily on who I work with. If someone asks me: is this 5,000 years or 10,000 years BP? I would even date enamel for them if there was no protein preserved, so long as I know they will either publish the limitations of the enamel method appropriately or include me as a co-author. If they just want a ‘number’, or I suspect that they will publish the date as a number, I will not date enamel for them. In terms of the reliability of XAD purification, my view is that it is not appropriately tested yet, rather than ‘I am unsure’. I am sure about my view. I also don’t think about contaminants as a homogenous group. I think about the type of contamination. So, yes, I would recommend (and do recommend) people submit samples contaminated with PVA [PVA jargon revised by Brock,Dee,Hughes,Snoeck,Staff and Bronk Ramsey [8]] (and other substances) to another lab for hydroxyproline isolation. If the sample is contaminated with a collagen glue, this would be pointless. If it just a little humic, then ultrafiltration is fine. | Accuracy  Alkali  *A priori* assessment  Caveat  Conservation substance  Pretreatment merit  Sample specificity |
| **48** | The use of the word ‘contamination’ in this survey does not accurately reflect diagenetic processes that result in the degradation of collagen without any contamination per se. | Depositional context |
| **50** | We’ve always used only ^14^C labs that pretreat the samples, or pre-treated in our own lab before sending for analysis. This is collagen extraction with ultrafiltration (30kD) at minimum. If I suspected carbon contamination, I wouldn’t submit the sample, or would treat it to remove it. For example, there were a few bones I knew were treated with consolidant. We took a sample from an area that I thought would have limited consolidant penetration, like the interior of a tooth root or tusk. Then the sample would be washed many times with acetone, then water. Then finally, what were the geochemical results? Was the C:N ratio off or the d13C value really different from other similar samples that might hint at whether the date was influenced by the consolidant? If there was any concern of this, the specimen was generally re-dated to confirm results. | ^14^C laboratory  Conservation substance  Pretreatment merit  Quality indicator  Sample specificity |
| **51** | I have dated many bones. I would only trust XAD and hydroxyproline extractions to provide reliable ages free of contaminants. I have seen all other methods fail. I tell my classes to consider ages derived on bone to be minimum ages (for the most part) unless they are XAD or HYP extractions. Better to pay more and get only one age that is reliable, than to spend the same amount for three ages on less reliable chemical fractions. | ^14^C laboratory  Pretreatment merit |
| **52** | I was head of two ^14^C labs, so I never submitted bones to another lab for pretreatment. | ^14^C laboratory |
| **54** | I only submitted samples that I thought were good (not contaminated) and I submitted them to a lab with a good reputation in the field, so I didn’t focus on the pretreatment specifically. | *A priori* assessment  ^14^C laboratory  Sample specificity |
| **56** | I have to confess that so far I more or less relied on the advice of collaborators that have more experience with regards to sample preparation of ^14^C samples. As faculty at a University of California campus I normally submit all samples to the ^14^C lab at UC Irvine [https://sites.uci.edu/keckams], of which also many people that know more about isotopes told me that it is a good lab. However, if their pretreatment is the best I can’t really say. | ^14^C laboratory |
| **64** | Each bone sample is unique with different levels of preservation and contamination. Unfortunately, there is no opportunity to treat every bone individually thus common methods and techniques have to be applied for bones of different state. Sometimes, this decreases the accuracy and precision of ^14^C dating but it is still time and cost-effective. | Accuracy  ^14^C laboratory  Sample specificity |
| **67** | Thank you for this interesting study, although I was surprised not to find any question related to quality indicators, which can be applied to collagen or other fractions of bone prepared for ^14^C dating from a given sample. The case is that for a good bone all preparation methods will give good results, and the simple extraction is sufficient and hydroxyproline extraction might be just a waste of work. The quality of dated material can be assessed in many ways (collagen yield, %N, %C, C:N, FTIR [https://en.wikipedia.org/wiki/Fourier-transform_infrared_spectroscopy], etc.) —so maybe a question like ‘How important is that any quality indicator is provided by ^14^C lab?’ should also be included. Without proper care, each more sophisticated preparation step may add contamination. | Pretreatment merit  Quality indicator  Sample specificity  Survey importance |
| **69** | My major expertise is geochemistry (including geochronology applied to palaeoclimatology, archeology and modern carbon cycle) so not easy to only select a sole answer in question 1.1 (Research discipline) but, as question 1.2 is restricted ^14^C dating of bone, I selected archaeology even if I mostly work on vegetal, sediment and soil organic matter, and specific molecules. The first question that arises to me is ‘what’s kind of contamination do I likely face?’, that’s the way to define which protocol would be the best! There is no miracle, ‘adaptation’ is the key word for ^14^C dating. Very often, if a bone is contaminated, I recommend the ninhydrin treatment, which focuses on only carbon imbedded in molecules, but this option was not available. One important criterion before choosing the right pretreatment is also C:N ratio and IR [https://en.wikipedia.org/wiki/Infrared_spectroscopy] analysis. | *A priori* assessment  Pretreatment merit |
| **77** | While most pretreatment methods seem to produce statistically consistent results, I’m beginning to use more refined methods (e.g., XAD purification) to confirm significant ages produced under different pretreatment protocols (e.g., ultrafiltration). | Accuracy  Pretreatment merit |
| **85** | Interesting — if you can improve the decision-making process for researchers who do not always work in ^14^C labs, great. I rely on a few ^14^C labs based on specialists I trust, and the problems I am aware of (ultrafiltration can still be problematic) and have directly experienced. If a new sample is known to be contaminated I wouldn’t voluntarily use it. ^14^C laboratories should have accurate error margins incorporated into their lab results. | *A priori* assessment ^14^C laboratory  Survey importance |
| **90** | I work with relatively recent material (almost always Holocene) and therefore I am not as sensitive to background correction issues as anyone working closer to the upper limit of ^14^C dating, but this is an important topic overlooked by the survey. On the other hand, I often have to work with artificially contaminated material (e.g., bone artefacts from museum collections, which were consolidated for conservation purposes), and my approach (like others’) is to remove as much of the consolidant as possible by physical and chemical methods before starting collagen extraction, as well as using spectroscopic methods to identify the contaminant and check whether it is still present in the extract. These steps are taken for granted in the survey, but are probably more important than the choice of extraction method. My lab is developing a ninhydrin-based protocol, which is a practical compromise between bulk collagen and single amino-acid dating. Although our work is unpublished, there are earlier publications in this area which are worth looking into. Almost any pretreatment method will give the right answer if the collagen content is good enough. Traditional acid-base-acid extraction is still favoured in many ^14^C labs because there is little opportunity to contaminate the sample in the lab. I know of situations where, for example, I suspect contamination from ultrafilters. For important samples, where cost and sample size are not the main constraints, I would attempt to date different chemical or molecular weight fractions of the same sample, and or date it by different extraction methods or in different labs, rather than trying to find a single gold-standard approach. | Alkali  ^14^C laboratory  Conservation substance  Pretreatment merit  Sample specificity |
| **91** | This survey seems to target the most accurate methods, like hydroxyproline extraction, but which also have lower yields with degraded collagen. Meanwhile, ultrafiltration is an easy method with reasonable yield-suiting most samples. | Pretreatment merit |
| **93** | One option that was not available in the list of priorities when choosing a pretreatment was EXPECTED AGE of the sample (rather than geographical location or species). This is important for my decision-making: if I am studying extinct megafauna whose remains may date near the limits of the ^14^C method, I would be much more likely to use a single amino-acid method than if I am studying late Prehistoric animals, where a small amount of modern carbon contamination would not make as much difference. | Sample specificity |
| **95** | I have had funding for and published probably less than 20 ^14^C dates, so my knowledge of methods and reliability is low. | Caveat |
| **98** | I think this survey is very significant for ^14^C dating of skeletal samples, we need more focus on pretreatment methods of bone, tooth or ivory samples for ^14^C dating. The issue of choosing the effective pretreatment method is always on the way, we need more communication to improve technology and methods, and the content of this survey can be helpful for researchers and engineers in ^14^C labs. | Pretreatment merit  Survey importance |
| **99** | Question 2.2 (and to some degree question 2.1) are misleading. When discussing contamination, it matters what type of contamination we are talking about. In general, hydroxyproline dating is the most reliable but, if the sample is contaminated with bone glue, then this is no longer the case, as bone glue also contains hydroxyproline. Also, setting up a functioning hydroxyproline system is challenging, as it has a higher risk of introducing exogenous carbon, which has to be thoroughly accounted for. Alternatively, ultrafiltration has a high reliability, but if the contaminant is of high molecular weight (e.g. certain conservation materials used in museums), then it has the opposite effect as it concentrates the contaminant. In this case hydroxyproline is better. Furthermore, if contamination is on the surface, then drilling the interior will do more than any pre-treatment. So, a universal ranking is difficult to achieve. Question 3.2 is also misleading — by asking whether a sample is contaminated by exogeneous carbon, people might talk about different things. Every bone, ivory, tooth sample contains exogenous carbon. The question is where and in what form. If it is dirt on the outside, it can easily be washed. If it is carbonates in the mineral component of the bone, any pre-treatment targeting collagen will do the trick. So, the question is more whether routine chemistry (e.g. acid-base-acid washes followed by Longin [29]’s suggested gelatinization) is sufficient. Both above: furthermore, we need to consider what time period we are trying to date. Contamination with younger carbon can be a tremendous problem for Palaeolithic samples, especially older than 30k BP. Many younger periods do not need the same rigorous treatment. Exceptions are periods where high precision is required but the calibration curve contains many short-lived wiggles. But in the latter case, wiggle matching may be more effective than pre-treatment adjustments. As I had to make a decision on what period I refer to (as they will lead to different conclusions in ranking the pre-treatment strategies), so I responded to questions 2.1 and 2.2 (Pretreatment) assuming a Palaeolithic sample older than 25,000 years BP with no glue contamination. | Alkali  *A priori* assessment  Caveat  Conservation substance  Pretreatment merit  Sample specificity |
| **100** | This survey doesn’t really seem tailored to ^14^C researchers such as myself. I am not sure that my answers are useful. | Caveat |
| **101** | The aim of the survey appears to be to decide which of three given treatments gives the most reliable results in an ideal world. As a colleague once said: *Good bones will give good dates in most cases, bad bones will often give bad dates*, no matter what you try. Reality is that one rarely has 250 grams (g) of bone material available for ^14^C dating. Either there is only very little bone, or they are bone artefacts of cultural importance that should sustain minimal damage in sampling. Furthermore, bone preservation is often modest or poor with only a few percent collagen remaining. Yet, ^14^C dating with AMS [https://en.wikipedia.org/wiki/Accelerator_mass_spectrometry] can give good results using 50 mg (good) to 500 mg (moderately poor) bone. The wise decision is to contact a reputable ^14^C-dating laboratory before sending any samples to discuss with them the best samples to select. This depends on the sample material available for dating, the contaminants to be expected based on field observations during excavation or conservation logs of museum items, the preservation of the bone/tooth/ivory, and the expertise of the ^14^C laboratory for the particular type of sample. To establish a reliable sample age, it may be advisable to date several fractions of one sample. The focus of the questionnaire only on three different final pretreatment steps neglects all the work leading up to these steps that is needed to make them successful. Collaboration between submitter and dater should be emphasized. | *A priori* assessment  Conservation substance  ^14^C laboratory  Sample specificity |
| **105** | All of my responses apply specifically to bone collagen, not tooth or ivory. | Caveat |
| **108** | I have worked in ^14^C dating labs as part of my PhD with Chris Turney at the University of Wollongong [https://research.unsw.edu.au/people/professor-chris-turney], and Keith Fifield [https://physics.anu.edu.au/contact/people/profile.php?ID=138], Stewart Fallon [http://rses.anu.edu.au/people/academics/dr-stewart-fallon] and Richard Gillespie [https://researchers.anu.edu.au/researchers/gillespie-r] at the Australian National University (ANU). I was also interviewed for a technician job in the ANU lab as the lab was wanting to move routinely into dating bone. When I was doing this work, we contacted the Waikato Radiocarbon Dating Laboratory (run by Alan Hogg [https://sci.waikato.ac.nz/about-us/people/alanh]) and the Oxford Radiocarbon Accelerator Unit (run by Tom Higham [http://www.ox.ac.uk/news-and-events/find-an-expert/professor-tom-higham]) regarding ultrafiltration of collagen. Of the two rounds of ultrafiltration needed [see 26], we were told only the first round was really necessary, and not the second. | ^14^C laboratory  Pretreatment merit |
| **110** | As a researcher at a ^14^C laboratory, this survey was not totally applicable. | Caveat |
| **111** | With respect to the question about submitting samples to a ^14^C laboratory, I have submitted many samples but I have always pretreated them myself, sending only material for direct combustion. I did not feel that the possible answers to this question adequately fit my experience. | Caveat |
| **117** | The survey is generally OK, but it would be useful to be able to give not a single answer to questions, but to choose several options for the same question. | Caveat |
| **121** | I extract collagen for isotopic analysis. Before sending a sample to be dated, I extract collagen and check established quality criteria (atomic C:N, % of C and N in collagen) in the sample. Then I check if my results match the results of the ^14^C-dating laboratory. Even the delta13C and delta15N values are informative. I usually ask for an Acid-Base-Acid extraction. I do not like ultrafiltration because I do not trust the cleaning of the filters — I have had bad experiences with that. | Quality indicator |
| **122** | I have never submitted bone samples to another ^14^C lab. Although I have measured numerous bones over the years at SUERC ^14^C lab. | Caveat |
| **126** | I am basically a consumer, but I learn that I need to be more involved. The survey is important. | Survey importance |
| **127** | Hydroxyproline is common in other tissues, notably plant cell walls (extensins). None of the methods seem to understand the biophysics and biochemistry of collagen. None refer to the latest research into humic substances, nor seeming to understand the concept if iso[e]lectric points or cooperative melting (sigh). C:N ratio is a very powerful tool for assessing collagen quality, due to the unusually high glycine content; however, no one seems to be aware of the theoretical C:N ratio of native (3.1214) or NaOH prepared (3.1777) collagen (sigh). There is a method which can measure the collagen composition in extracts. It is called proteomics, however this in not mentioned in your survey. It is used in Brock,Dee,Hughes,Snoeck,Staff and Bronk Ramsey [8]. | Alkali  Caveat  Pretreatment merit  Quality indicator |

**References**

1. Beaumont W, Beverly R, Southon J, Taylor RE. 2010 Bone preparation at the KCCAMS laboratory. *Nuclear Instruments and Methods in Physics Research Section B: Beam Interactions with Materials and Atoms*. **268**, 906-909. (<https://doi.org/10.1016/j.nimb.2009.10.061>)

2. Becerra-Valdivia L, Waters MR, Stafford TW, Anzick SL, Comeskey D, Devièse T, Higham T. 2018 Reassessing the chronology of the archaeological site of Anzick. *Proc Nat Acad Sci*. **115**, 7000-7003. (<https://doi.org/10.1073/pnas.1803624115>)

3. Bird MI, Ayliffe LK, Fifield LK, R. C, Turney CSM, Barrows TT, David B. 1999 Radiocarbon dating of “old” charcoal using a wet oxidation-stepped combustion procedure. *Radiocarbon*. **41**, 127-140. (<https://doi.org/10.1017/S0033822200019482>)

4. Boudin M, Bonafini M, Van Den Brande T, Vanden Berghe I. 2017 Cross-flow nanofiltration of contaminated protein-containing material: state of the art. *Radiocarbon*. **59**, 1793-1807. (<https://doi.org/10.1017/RDC.2017.137>)

5. Brock F, Bronk Ramsey C, Higham T. 2007 Quality assurance of ultrafiltered bone dating. *Radiocarbon*. **49**, 187-192. (<http://doi.org/10.1017/S0033822200042107>)

6. Brock F, Higham T, Ditchfield P, Bronk Ramsey C. 2010 Current pretreatment methods for AMS radiocarbon dating at the Oxford Radiocarbon Accelerator Unit (ORAU). *Radiocarbon*. **52**, 103-112. (<https://doi.org/10.1017/S0033822200045069>)

7. Brock F, Geoghegan V, Thomas B, Jurkschat K, Higham TFG. 2013 Analysis of bone “collagen” extraction products for radiocarbon dating. *Radiocarbon*. **55**, 445-463. (<http://dx.doi.org/10.1017/S0033822200057581>)

8. Brock F, Dee M, Hughes A, Snoeck C, Staff R, Bronk Ramsey C. 2018 Testing the effectiveness of protocols for removal of common conservation treatments for radiocarbon dating. *Radiocarbon*. **60**, 35-50. (<https://doi.org/10.1017/RDC.2017.68>)

9. Brown TA, Nelson DE, Vogel JS, Southon JR. 1988 Improved collagen extraction by modified Longin method. *Radiocarbon*. **30**, 171-177. (<http://doi.org/10.1017/S0033822200044118>)

10. Bruhn F, Duhr A, Grootes PM, Mintrop A, Nadeau M-J. 2001 Chemical removal of conservation substances by “Soxhlet”-Type Extraction. *Radiocarbon*. **43**, 229-237. (<https://doi.org/10.1017/S0033822200038054>)

11. Cersoy S, Zazzo A, Lebon M, Rofes J, Zirah S. 2017 Collagen extraction and stable isotope analysis of small vertebrate bones: a comparative approach. *Radiocarbon*. **59**, 679-694. (<https://doi.org/10.1017/RDC.2016.82>)

12. Cook GT, Higham TFG, Naysmith P, Brock F, Freeman SPHT, Bayliss A. 2012 Assessment of Infinite-Age Bones from the Upper Thames Valley, UK, as 14C Background Standards. *Radiocarbon*. **54**, 845-853. (<https://doi.org/10.2458/azu_js_rc.v.16161>)

13. DeNiro MJ. 1985 Postmortem preservation and alteration of *in vivo* bone collagen isotope ratios in relation to palaeodietary reconstruction. *Nature*. **317**, 806-809. (<https://doi.org/10.1038/317806a0>)

14. Devièse T, Comeskey D, McCullagh J, Bronk Ramsey C, Higham T. 2017 New protocol for compound-specific radiocarbon analysis of archaeological bones. *Rapid Commun Mass Spectrom*. **32**, 373-379. (<https://doi.org/10.1002/rcm.8047>)

15. Devièse T, Stafford TW, Waters MR, Wathen C, Comeskey D, Becerra-Valdivia L, Higham T. 2018 Increasing accuracy for the radiocarbon dating of sites occupied by the first Americans. *Quat Sci Rev*. **198**, 171-180. (<https://doi.org/10.1016/j.quascirev.2018.08.023>)

16. Dinnis R, Pate A, Reynolds N. 2016 Mid-to-late Marine Isotope Stage 3 mammal faunas of Britain: a new look. *Proceedings of the Geologists’ Association*. **127**, 435-444. (<https://doi.org/10.1016/j.pgeola.2016.05.002>)

17. Dunbar E, Cook GT, Naysmith P, Tripney BG, Xu S. 2016 AMS ^14^C dating at the Scottish Universities Environmental Research Centre (SUERC) Radiocarbon Dating Laboratory. *Radiocarbon*. **58**, 9-23. (<https://doi.org/10.1017/RDC.2015.2>)

18. Dunbar E, Naysmith P, Cook GT, Scott EM, Xu S, Tripney BG. 2017 Investigation of the Analytical F14C Bone Background Value at SUERC. *Radiocarbon*. **59**, 1463-1473. (<https://doi.org/10.1017/RDC.2017.67>)

19. Gillespie R, Hedges REM, Wand JO. 1984 Radiocarbon dating of bone by accelerator mass spectrometry. *J Archaeol Sci*. **11**, 165-170. (<https://doi.org/10.1016/0305-4403(84)90051-7>)

20. Gilmour DM, Butler VL, O'Connor JE, Davis EB, Culleton BJ, Kennett DJ, Hodgins G. 2015 Chronology and ecology of late Pleistocene megafauna in the northern Willamette Valley, Oregon. *Quatern Res*. **83**, 127-136. (<https://doi.org/10.1016/j.yqres.2014.09.003>)

21. Higham TFG, Jacobi RM, Ramsey CB. 2006 AMS radiocarbon dating of ancient bone using ultrafiltration. *Radiocarbon*. **48**, 179-195. (<https://doi.org/10.1017/S0033822200066388>)

22. Higham T. 2011 European Middle and Upper Palaeolithic radiocarbon dates are often older than they look: problems with previous dates and some remedies. *Antiquity*. **85**, 235-249. (<https://doi.org/10.1017/S0003598X00067570>)

23. Hüls MC, Grootes PM, Nadeau M-J. 2007 How clean is ultrafiltration cleaning of bone collagen? *Radiocarbon*. **49**, 193-200. (<https://doi.org/10.1017/S0033822200042119>)

24. Hüls CM, Grootes PM, Nadeau MJ. 2009 Ultrafiltration: boon or bane? *Radiocarbon*. **51**, 613-625. (<https://doi.org/10.1017/S003382220005596X>)

25. Huels M, van der Plicht J, Brock F, Matzerath S, Chivall D. 2017 Laboratory Intercomparison of Pleistocene Bone Radiocarbon Dating Protocols. *Radiocarbon*. **59**, 1543-1552. (10.1017/RDC.2017.23)

26. Jacobi RM, Higham TFG, Ramsey CB. 2006 AMS radiocarbon dating of Middle and Upper Palaeolithic bone in the British Isles: improved reliability using ultrafiltration. *J Quat Sci*. **21**, 557-573. (<https://doi.org/10.1002/jqs.1037>)

27. Kosintsev PA, Mitchell KJ, Devièse T, van der Plicht J, Kuitems M, Petrova E, Tikhonov A, Higham T, Comesky D, Turney CRM*, et al.* 2018 Evolution and extinction of the “Siberian unicorn” *Elasmotherium sibiricum*, and late Quaternary megafaunal extinction before the Last Glacial Maximum. *Nature Ecology & Evolution*. **3**, 31-38. (<https://doi.org/10.1038/s41559-018-0722-0>)

28. Kuzmin YV. 2019 The older, the better? On the radiocarbon dating of Upper Palaeolithic burials in Northern Eurasia and beyond. *Antiquity*. **93**, 1061-1071. (<https://doi.org/10.15184/aqy.2018.158>)

29. Longin R. 1971 New method of collagen extraction for radiocarbon dating. *Nature*. **230**, 241-242. (<https://doi.org/10.1038/230241a0>)

30. Marom A, McCullagh JSO, Higham TFG, Sinitsyn AA, Hedges REM. 2012 Single amino acid radiocarbon dating of Upper Paleolithic modern humans. *Proc Nat Acad Sci*. **109**, 6878-6881. (<https://doi.org/10.1073/pnas.1116328109>)

31. McDonald HG, Stafford TW, Gnidovec DM. 2015 Youngest radiocarbon age for Jefferson's ground sloth, *Megalonyx jeffersonii* (Xenarthra, Megalonychidae). *Quatern Res*. **83**, 355-359. (<https://doi.org/10.1016/j.yqres.2014.11.006>)

32. Politis GG, Messineo PG, Stafford TW, Lindsey EL. 2019 Campo Laborde: a Late Pleistocene giant ground sloth kill and butchering site in the Pampas. *Science Advances*. **5**, eaau4546. (<https://doi.org/10.1126/sciadv.aau4546>)

33. Potter BA, Reuther JD, Newbold BA, Yoder DT. 2012 High resolution radiocarbon dating at the Gerstle River Site, Central Alaska. *Am Antiq*. **77**, 71-98. (<http://dx.doi.org/10.7183/0002-7316.77.1.71>)

34. Redmond BG, McDonald HG, Greenfield HJ, Burr ML. 2012 New evidence for Late Pleistocene human exploitation of Jefferson's Ground Sloth (*Megalonyx jeffersonii*) from northern Ohio, USA. *World Archaeol*. **44**, 75-101. (<https://doi.org/10.1080/00438243.2012.647576>)

35. Stafford TW, Hare PE, Currie L, Jull AJT, Donahue DJ. 1991 Accelerator radiocarbon dating at the molecular level. *J Archaeol Sci*. **18**, 35-72. (<https://doi.org/10.1016/0305-4403(91)90078-4>)

36. Tisnérat-Laborde N, Valladas H, Kaltnecker E, Arnold M. 2003 AMS radiocarbon dating of bones at LSCE. *Radiocarbon*. **45**, 409-419. (<https://doi.org/10.1017/S003382220003277X10.1017/S003382220003277X>)

37. van Klinken GJ. 1999 Bone collagen quality indicators for palaeodietary and radiocarbon measurements. *J Archaeol Sci*. **26**, 687-695. (<https://doi.org/10.1006/jasc.1998.0385>)

38. Waterbolk HT. 1971 Working with radiocarbon dates. *Proceedings of the Prehistoric Society*. **37**, 15-33. ( <https://doi.org/10.1017/S0079497X00012548>)

39. Waters MR, Stafford TW. 2014 Redating the Mill Iron Site, Montana: a reexamination of Goshen complex chronology. *Am Antiq*. **79**, 541-548. ( <https://doi.org/10.7183/0002-7316.79.3.541>)

40. Waters MR, Stafford TW, Kooyman B, Hills LV. 2015 Late Pleistocene horse and camel hunting at the southern margin of the ice-free corridor: Reassessing the age of Wally’s Beach, Canada. *Proc Nat Acad Sci*. **112**, 4263-4267. (<https://doi.org/10.1073/pnas.1420650112>)

41. Zazula GD, MacPhee RDE, Metcalfe JZ, Reyes AV, Brock F, Druckenmiller PS, Groves P, Harington CR, Hodgins GWL, Kunz ML*, et al.* 2014 American mastodon extirpation in the Arctic and Subarctic predates human colonization and terminal Pleistocene climate change. *Proc Nat Acad Sci*. **111**, 18460-18465. (<https://doi.org/10.1073/pnas.1416072111>)
